# Identification of novel human adenovirus candidates using the coxsackievirus and adenovirus receptor for cell entry

**DOI:** 10.1186/s12985-020-01318-w

**Published:** 2020-04-09

**Authors:** Kemal Mese, Oskar Bunz, Sebastian Schellhorn, Wolfram Volkwein, Dominik Jung, Jian Gao, Wenli Zhang, Armin Baiker, Anja Ehrhardt

**Affiliations:** 1 Institute for Virology and Microbiology, Center for Biomedical Education and Research (ZBAF), Witten/Herdecke University, Stockumer Str. 10, 58453 Witten, Germany; 2 Bavarian Health and Food Safety Authority (LGL), Oberschleissheim, Germany

**Keywords:** Adenovirus, Luciferase, Virus library, Receptor, CAR

## Abstract

**Background:**

There are over 100 known human adenovirus (HAdV) types, which are able to cause a broad variety of different self-limiting but also lethal diseases especially in immunocompromised patients. Only limited information about the pathogenesis and biology of the majority of these virus types is available. In the present study, we performed a systematic screen for coxsackievirus and adenovirus receptor (CAR)-usage of a large spectrum of HAdV types.

**Methods:**

To study receptor usage we utilized a recombinant HAdV library containing HAdV genomes tagged with a luciferase and GFP encoding transgene. We infected CHO-CAR cells stably expressing the CAR receptor and to much information with tagged viruses (HAdV3, 14, 16, 50, 10, 24, 27, 37 and 69) and measured luciferase expression levels 26 and for some viruses (AdV10, − 24 and − 27) 52 h post-infection. As positive control, we applied human adenovirus type 5 (HAdV5) known to use the CAR receptor for cell entry. For viruses replication studies on genome level we applied digital PCR.

**Results:**

Infection of CHO-CAR and CHO-K1 cells at various virus particle numbers per cell (vpc) revealed that HAdV10, 24, and 27 showed similar or decreased luciferase expression levels in the presence of CAR. In contrast, HAdV3, 14, 16, 50, 37 and 69 resulted in increased luciferase expression levels in our initial screening experiments. CAR usage of HAdV3, 14, 50, and 69 was not studied before, and therefore we experimentally confirmed CAR usage for these HAdV as novel viruses utilizing CAR as a receptor. To rule out that replication of HAdV in transduced CHO cells is responsible for increased transduction rates we performed replication assays on virus genome level, which revealed that there is no HAdV replication.

**Conclusion:**

In the present study, we screened a HAdV library and identified novel human HAdV using the CAR receptor. To our knowledge, this is the first description of CAR usage for HAdV 3, 14, 50, and 69.

## Main text

In the clinic, human adenoviruses (HAdV) gained increasing importance. They cause different clinical symptoms with a wide range of diseases such as conjunctivitis, gastroenteritis, pneumonia, and myocarditis. Most threatened groups are children younger than 5 years and immune-deficient patients for instance after transplantation. In the US-military bases HAdV caused pneumonia outbreaks were reported [1]. Until now over 100 HAdV were identified (http://hadvwg.gmu.edu/) which are divided into six species (A-G). Adenoviruses have a size of 65 to 85 nm in diameter and they belong to the group of non-enveloped viruses. The capsid consists of 252 capsomeres with an icosahedral shape. It is comprised of 240 hexon trimers, 12 pentons and 12 fiber proteins protruding from the penton base. The genome of HAdV is a linear double-stranded DNA which is approximately 26–46 kbp in length dependent on the adenovirus type [1]. Adenoviruses are known as pathogens in the clinic but they are also explored as viral vectors in gene therapeutic applications. Historically, predominantly HAdV5 was investigated as viral vector, but it became clear over the past decade that this virus type displays limitations associated with its seroprevalence and tropism. Towards that end other than HAdV5 adenovirus types were explored as gene therapeutic agent. However, it is crucial to further understand biological features of these viruses to pursue them in preclinical and clinical studies. This includes the virus tropism to achieve an effective therapy to cure viral infections but also to develop improved vectors for gene therapeutic applications [2, 3].

It was shown that for attachment and cell entry HAdV binds to CD46, heparansulfate, sialic acid, integrins, CD80/86, desmoglein 2, and CAR presented on the cell surface [4–11]. CAR is a 46 kda protein which belongs to the Immunoglobulin (Ig) superfamily and possesses two extracellular immunoglobulin-like domains. The tissue distribution is not completely understood. Biodistribution analyses on the level of mRNA revealed that mRNA is present in different organs like brain, heart, intestine, pancreas, lung, liver and kidney. There is evidence for CAR-mediated virus attachment for HAdV 2, 4, 5, 9, 12, 19, 31, 37, and 41 [12, 13]. However, these studies represent punctual studies and therefore we aimed at studying CAR usage of a broad spectrum of human adenoviruses derived from different species.

We took advantage of a luciferase and GFP tagged HAdV library, which was generated in our laboratory [14]. The generated viruses are replication-competent and contain a monocistronic luciferase and GFP expression cassette in the adenovirus early region E3, which allows measuring and visualization of adenovirus transduction efficiencies. After transduction of cells, luciferase and GFP expression levels directly correlate with adenovirus transduction efficiencies. Here we screened HAdV3 (species B1), 16 (species B1), 50 (species B1), 14 (species B2), 10 (species D), 24 (species D), 27 (species D), 37 (species D), and 69 (species D) for CAR usage. Note that for HAdV3, 14, 16, and 50 it was demonstrated that these viruses utilize CD46 for cell entry and in addition to CD46 HAdV3 and all members of HAdV species B can utilize CD80/86 ([10]. HAdV37 was shown to bind to CD46, sialic acid and CAR [15, 16]. To our knowledge, there is no information on cell attachment and binding factors for HAdV 10, 24, 27, and 69. Note that for HAdV5 it is well established that this virus utilizes CAR for cell entry, and therefore it was applied as a positive control in the present study.

**Fig. 1:**
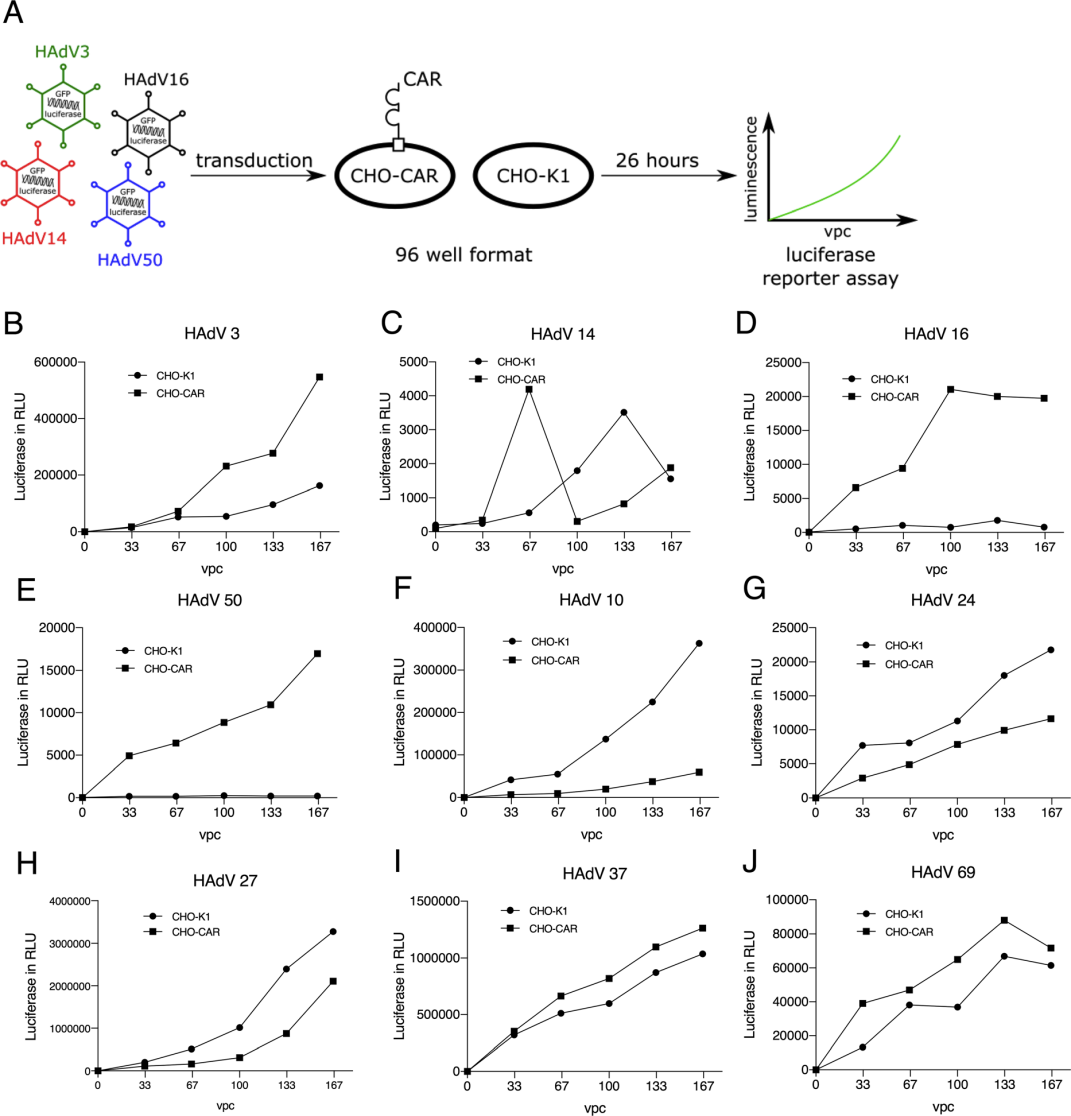
**Schematic visualization of the screening procedure (a) and results of screening reporter-gene tagged species B and D adenoviruses on CHO-CAR and CHO-K1 cells (b-j).** Cells were infected at various viral particle numbers per cell (vpc) and luciferase expression levels were measured 26 h post-infection. HAdV3, 14, 16, 50, 10, 24, 27, 37 and 69 **(b-j)** were analyzed. These experiments were performed in triplicates, which were pooled for measurement. RLU: relative light units

Here we explored CHO-CAR cells stably expressing the human coxsackievirus and adenovirus receptor and CHO-K1 CAR-negative control cells, which were cultured in DMEM medium (PAN-Biotech GmbH, Aidenbach, Germany) with 10% FCS (PAN-Biotech GmbH, Aidenbach, Germany) and 1% Penicillin/Streptomycin (PAN-Biotech GmbH, Aidenbach, Germany) using 5% CO_2_ at 37 °C. For selection, we added 100 μl G418 (50 mg/ml) to the culture medium of both CHO cell -lines. Cells were seeded at a density of 3 × 10^4^ per well in 96-well tissue culture plates in triplicates for each test. After infection with respective total virus particle numbers per cell (vpc), all luciferase measurements were performed 26 h post-infection **(**Fig. 1a**)**.

**Fig. 2:**
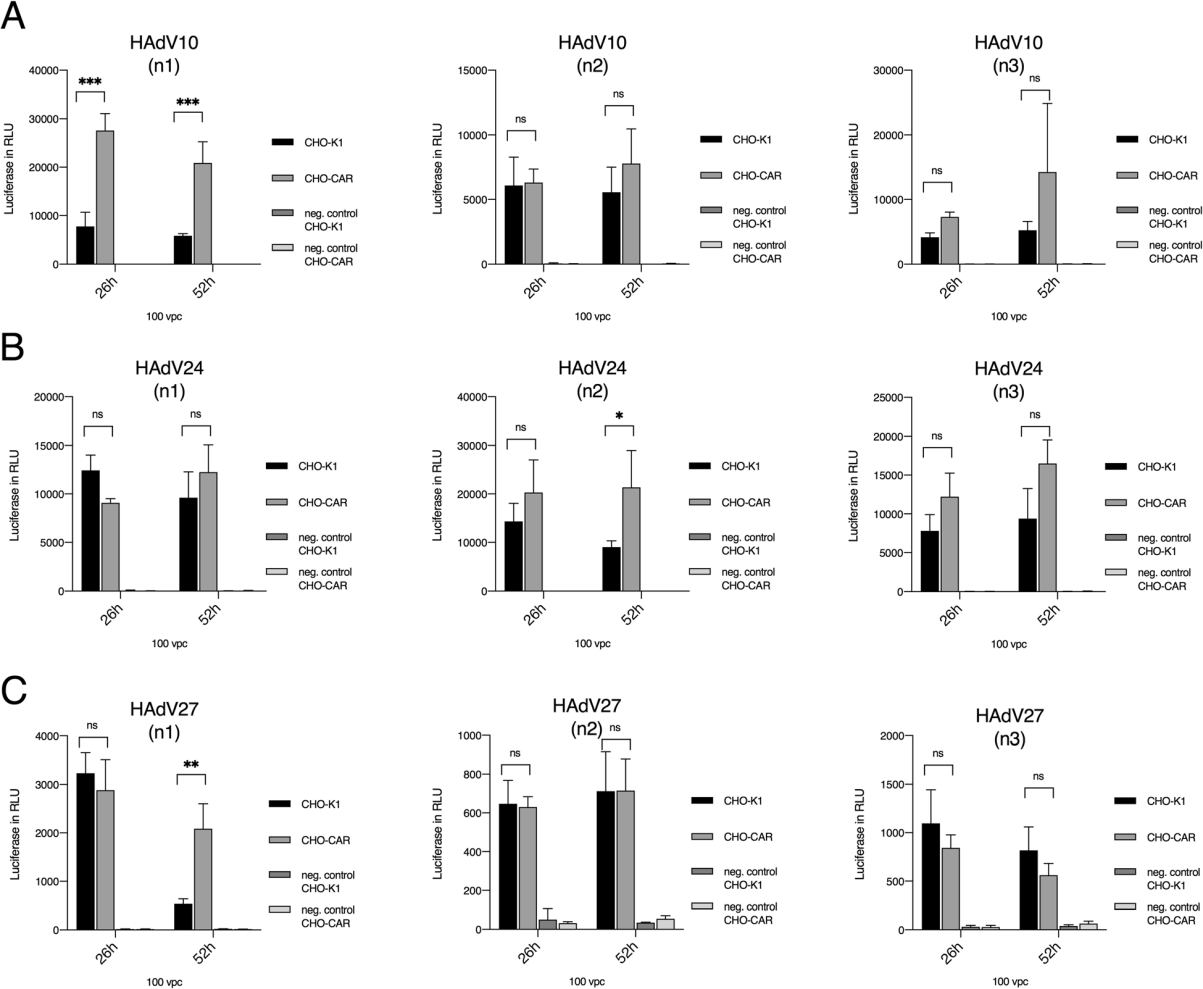
**Screening of HAdV10, 24 and 27 luciferase expressions in CHO-CAR and CHO-K1 at two different time points.** Cells were infected with 100 vpc and harvested at two different time points (26 and 52 h post-infection) with HAdV10 **(a)**, HAdV24 **(b)**, HAdV27 **(c)**. Each experiment was performed in biological and technical triplicates. RLU: relative light units. For statistical analyses a two-way ANOVA was performed. Displayed are means + standard deviation. * a-values ≤ 0.05, **, ≤ 0.005*** ≤ 0.0005

Initially we performed a first screening based on infection of CHO-CAR and CHO-K1 cells utilizing all available viruses. Cells grown in a 96-well plate were infected with 33, 67, 100, 133, 167 vpc per well and luciferase assays were performed. For HAdV derived from species B, HAdV3, HAdV16, HAdV50 and HAdV14 showed increased virus uptake after infection of CHO-CAR cells in comparison to CHO-K1 cells (Fig. 1**b-e**). For species D viruses, HAdV37 and 69 showed increased uptake into CHO-CAR cells if directly compared to CHO-K1 cells as measured by luciferase values 26 h post-infection (Fig. 1**i-j**). In contrast, after infection with HAdV10, 24, and 27 we measured even lower luciferase values compared to control cells (CHO-K1), demonstrating that these viruses fail to use the CAR receptor for cellular uptake (Fig. 1**f-h**). With these viruses we performed luciferase measurements at two different time-points (26 and 52 h after infection) using 100 vpc, to address the question whether virus attachment and uptake may take longer for these viruses. However, this hypothesis could not be confirmed **(**Fig. 2**a-c)** and no conclusive statements could be obtained. Table 1 summarizes results for all screened viruses in these initial screening experiments.

**Table 1:** Summary of tested viruses for CAR receptor usage in the present study. (−) No higher luciferase measurements on CHO-CAR cells;(+) Low but significant higher luciferase measurements; (++) Higher luciferase measurements

HAdV (species)	CAR receptor usage
3 (B1)	**+**
16 (B1)	**+**
50 (B1)	**++**
14 (B2)	**+**
5 (C)	**++**
10 (D)	**+**
24 (D)	**+/−**
27 (D)	**–**
37 (D)	**+**
69 (D)	**++**

**Fig. 3:**
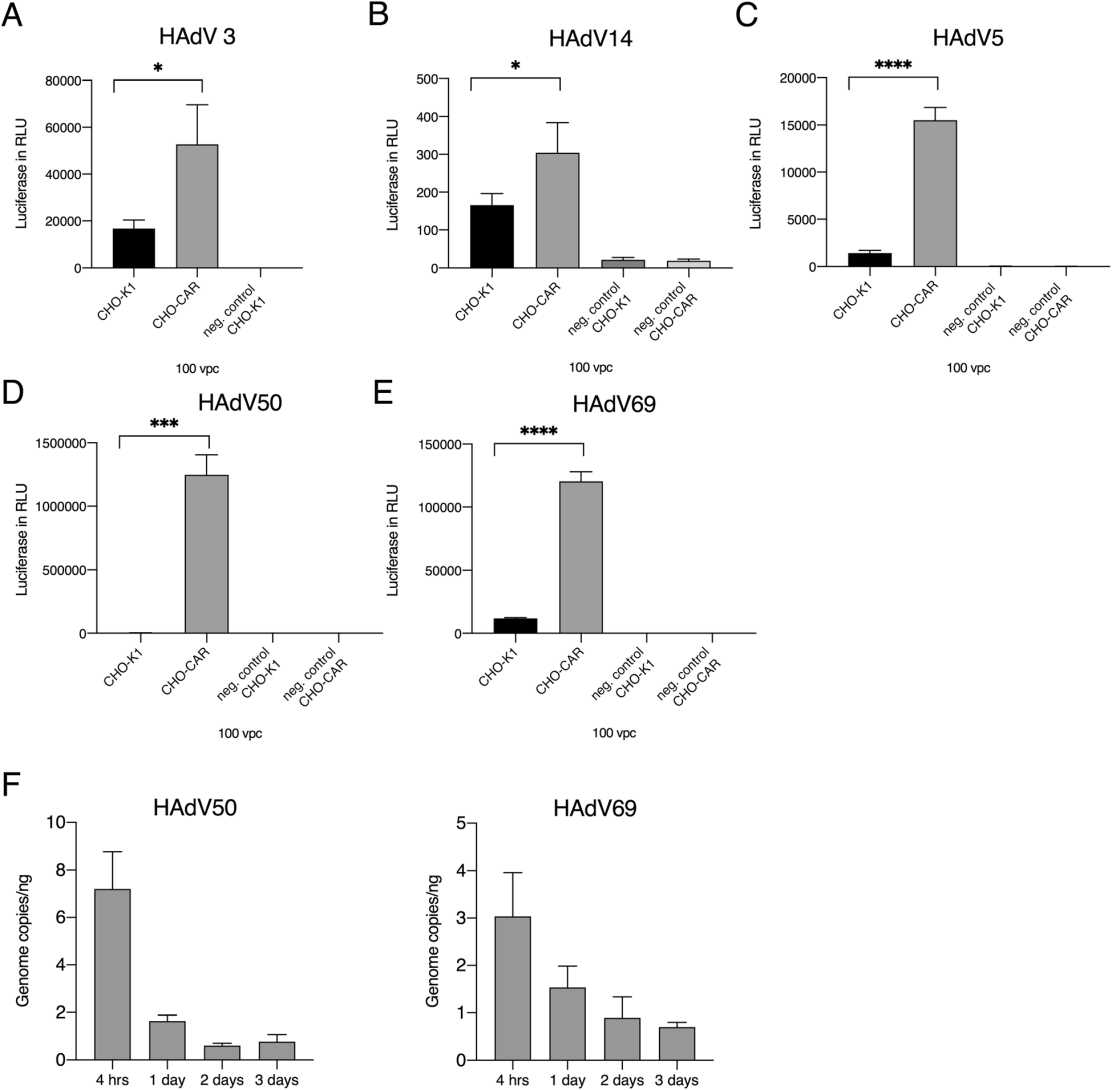
**Infection efficiencies for HAdV5, 3, 14, 50 and 69 on CHO-CAR and CHO-K1 control cells and quantification of HAdV50 and HAdV69 vector genomes over time in CHO-CAR cells.** Cells were infected with HAdV3 **(a)**, HAdV14 **(b)**, HAdV5 **(c)** and HAdV50 **(d)** and HAdV69 **(e)** at 100 viral particle numbers per cell (vpc) and luciferase expression levels were measured 26 h post-infection. As positive control, HAdV5 was applied and uninfected cells were measured referring to the negative control. For each virus, experiments were performed in biological and technical triplicates. **(f)** Quantification of HAdV50 and HAdV69 vector genomes in CHO-CAR cells after 4 h, 1 day, 2 days and 3 days after infection with 100 vpc using a digital droplet PCR approach. For statistical analyses a student’s t-test was performed. Displayed are means + standard deviation. * *p*-values ≤ 0.05, ** ≤ 0.005

For further experiments, we selected out HAdV14, 50, 3 and 69 for which the information on receptor usage is scarce. These viruses displayed increased cellular uptake in the presence of the CAR receptor (Figs. 3**a-e**). In the following steps, we applied 100 vpc of these viruses and performed biological and technical triplicates. As positive control we used AdV5 because it is well established that this virus utilizes CAR as primary receptor. We found that all viruses resulted in significantly increased luciferase expression levels 26 h after infection (Fig. 3**a-e**). Since the HAdV69 and 50 showed extremely high luciferase values 26 h post-infection, we addressed the question whether these human adenoviruses may replicate in Chinese hamster ovarian (CHO) cells, despite the species barrier. To address this question we performed a replication study in CHO-CAR cells by quantifying viral vector genomes 4 h, 1 day, 2 days and 3 days post-infection with 100 vpc. Detection of increasing vector genome copy numbers over time would be a strong indicator for virus replication in CHO cells. To quantify vector genome copy numbers we used a droplet digital PCR approach.

Genomic DNA was purified according the instructions of the producer (NucleoSpin®, MACHEREY-NAGEL, Germany). For the detection of genome copy numbers of HAdV50 and HAdV69, droplet digital PCR (ddPCR) using primers Ad1 (5′-GCC ACG GTG GGG TTT CTA AAC TT-3′) and Ad2 (5′-GCC CCA GTG GTC TTA CAT GCA CAT C-3′) according to Heim et al. [17] was performed. In order to meet the criteria for ddPCR, the respective probe was slightly modified to Ad_ddPCR_probe (5′6FAM-TGC ACC AGA /ZEN/ CCC GGG CTC AGG TAC TCC GA 3’IABkFQ). The total ddPCR reaction volume was 20 μL, containing 10 μL of ddPCR supermix for probes (Bio-Rad, Munich, Germany), 400 nM of primers Ad1 and Ad2, 500 nM of probe Ad_ddPCR_probe, and 2 μL of extracted DNA. Droplets were generated using 70 μL of droplet generation oil (Bio-Rad, Munich, Germany) in a QX100 droplet generator (Bio-Rad,Munich, Germany) and then transferred to a 96-well plate which was heat-sealed afterwards. PCR reaction was performed in a T100 PCR thermocycler using the following temperature profile: 95 °C for 10 min, 60 cycles at 94 °C for 30 s and 59 °C for 2 min, 98 °C for 10 min. For all steps, a ramp rate of 1 °C/second was used. Afterwards, droplets were analyzed with the QX100 droplet reader (Bio-Rad, Munich, Germany) in combination with Quantasoft software, version 1.7.4.0917 (Bio-Rad, Munich, Germany) and the results were normalized to 1 ng of extracted DNA. As displayed in Fig. 3f no vector genome replication is detectable in CHO-CAR cells and therefore, we could exclude the replication of HAdV50 and 69 in CHO cells **(**Fig. 3f**)**. We can only speculate for the reasons of high transgene expression levels of HAdV69 and HAdV50. Possibly more vector genome copy numbers might enter the nucleus (as compared to other serotypes) which then leads to increased transgene expression levels.

In summary in our work, we applied a luciferase/GFP-tagged virus library to study the usage of CAR as an entry receptor for adenoviruses. Luciferase measurements revealed usage of CAR for HAdV3, 16, 14, 50 and 69, which was not shown before. Interestingly, we observed lower luciferase expression levels in CHO-CAR cells for HAdV10, 24 and 27, if directly compared to CHO-K1 cells lacking the receptor. Further investigations are needed to shed light on this phenomenon. However, we speculate that there could be a possible blocking effect of CAR mediated by binding of the virus to the cellular surface without uptake into the cell. Potentially CAR only captures the virus but cellular import or downstream processes related to virus trafficking into the nucleus may be impaired. As described previously HAdV37 can bind to CAR, but seems to be less important for virus uptake, which is in concordance with the literature showing the binding ability of HAdV37 to CAR-D1 [8, 16]. Note that for HAdV14 there are conflicting results regarding the direct comparison virus uptake into CHO-CAR and CHO-K1 control cells (Fig. 1c and Fig. 3b) at 100 vpc per cell. Note that there are studies describing reduced CAR expression on tumor cells [18, 19] and also reduced CAR expression on infected hematopoietic cells [20]. In the context of these findings we believe that results presented in this study utilizing CHO cells overexpressing CAR are needed, because they represent an initial screening tool to analyze receptor usage of adenoviruses.

## Data Availability

Further information and material published in this study can be obtained on request.

## Abbreviations

CAR: Coxsackievirus- and adenovirus receptor
CHO cells: Chinese hamster ovarian cells
ddPCR: Droplet digital PCR
FBS: Fetal bovine serum
GFP: Green fluorescent protein
HAdV: Human adenovirus
kbp: Kilo base pairs
kDa: Kilo Dalton
RLU: Relative light units
